# Association between serum pyridoxal 5′-phosphate levels and chronic obstructive pulmonary disease: findings based on NHANES 2005–2010

**DOI:** 10.3389/fnut.2024.1526575

**Published:** 2025-01-10

**Authors:** Yanbin Liu, Jun Yan

**Affiliations:** ^1^Department of Respiratory, Dongzhimen Hospital, Beijing University of Chinese Medicine, Beijing, China; ^2^Department of Respiratory, The Third Affiliated Hospital, Beijing University of Chinese Medicine, Beijing, China

**Keywords:** COPD, vitamin B6, serum pyridoxal 5′-phosphate, PLP, NHANES

## Abstract

**Objective:**

Vitamin B6 is involved in regulating a variety of biological reactions in cell metabolism, and possesses antioxidant and anti-inflammatory biological functions. 5′-pyridoxal phosphate (PLP) is the main biologically active form of vitamin B6. There is currently no research on the correlation between serum PLP levels and chronic obstructive pulmonary disease (COPD) prevalence. This study aims to explore the relationship between serum PLP levels and COPD prevalence.

**Methods:**

This cross-sectional study included adult participants with complete data on COPD diagnosis and serum PLP levels from 2005 to 2010 National Health and Nutrition Examination Survey (NHANES) database. Generalized linear model (GLM) was employed to analyze the association between serum PLP levels and COPD prevalence. The restricted cubic spline (RCS) curve and threshold effect analysis were used to explore the potential non-linear relationship between serum PLP levels and COPD prevalence. Subgroup analysis was carried out to assess the robustness of the relationship between serum PLP levels and COPD prevalence.

**Results:**

A total of 11,103 participants were included in this study, of whom 830 were diagnosed with COPD. Higher PLP levels are associated with a reduced prevalence of COPD. GLM analysis confirmed that the groups with higher PLP levels (Q3 and Q4) had a significantly lower prevalence rate of COPD compared to the group of Q2. The RCS curves showed a non-linear “L”-shaped relationship between serum PLP levels and COPD prevalence. The threshold effect analysis found a critical point of 43.3 nmol/L. When serum PLP level is below 43.3 nmol/L, there is a negative correlation between serum PLP levels and COPD prevalence (*p* for overall <0.001, *p* for nonlinear <0.001). Subgroup analysis and interaction tests confirmed the robustness of the relationship.

**Conclusion:**

This study is the first to discover a non-linear relationship between serum PLP levels and COPD prevalence. Higher serum PLP levels are associated with a reduced prevalence of COPD.

## Introduction

COPD is a chronic and progressive respiratory disease, which is characterized by recurrent coughing, sputum, and difficulty breathing (1). Its principal pathological changes include emphysema and airway stenosis. In case where lung tissue sustains severe damage, patients will encounter pulmonary dysfunction (2). In recent years, the global mortality rate of COPD has continued to rise, and it was reported to be the third leading cause of death worldwide in 2020 (3). COPD not only gives rise to substantial social and economic burdens, but also exerts a serious impact on the physical and mental health of patients (4, 5). Thus, it is of utmost significance to find effective prevention and treatment measures for COPD.

Vitamin B6, also known as pyridoxine, is a water-soluble vitamin (6). PLP is the main biologically active form of vitamin B6 in the human body and participates in many enzymatic reactions as a coenzyme (7). Vitamin B6 is an essential exogenous micronutrient for human body, with major food sources including meat, fish, and poultry (8). It is not only involved in regulating a variety of biological reactions in cell metabolism, but also has antioxidant and anti-inflammatory biological functions. It is crucial for the treatment of cardiovascular and cerebrovascular diseases, diabetes, cancer, neurological disease and other diseases. Meanwhile, vitamin B6 also has therapeutic effects in preventing and treating vitamin B6 deficiency, reducing vomiting during pregnancy, and treating seborrheic dermatitis. As an important nutrient, it is added to various foods such as grains and flour to enhance the nutritional value of food. Vitamin B6 is also one of the essential nutrients for animal growth and development, and is therefore added to feed to enhance animal immunity and stress resistance. Thereby, it is an essential nutrient that is highly demanded by pharmaceutical, feed, and food processing markets (9–12). The relationship between vitamin B6 and COPD complications is gradually drawing attention. Previous studies have indicated that COPD patients with a diminished intake of vitamin B6 have a greater susceptibility to frailty (13), and vitamin B6 deficiency is a potential thrombotic factor for COPD patients (14).

COPD mainly presents with airway remodeling and the infiltration of inflammatory cells such as neutrophils, CD8+ T lymphocytes and activated macrophages as the main pathological features. Inflammatory factors play a crucial and decisive role in the occurrence and the development of COPD. Oxidative stress is an important and prominent factor in the pathogenesis of COPD, participating in a variety of pathogenic processes such as lung cell damage and mucus secretion (15, 16). And vitamin B6 precisely has functions like regulating the production of inflammatory mediators, inhibiting the release of pro-inflammatory factors, modulating immune function and counteracting oxidative stress. The relationship between vitamin B6 and COPD prevalence is worthy of research (17).

Presently, there is a dearth of research concerning the correlation between serum PLP levels and COPD prevalence. This study adopts a cross-sectional research design, utilizing participants from the NHANES database from 2005 to 2010 as the research subjects, with the aim of probing into the relationship between serum PLP levels and COPD prevalence and providing further evidence for the prevention and treatment of COPD with vitamin B6.

## Methods

### Study design and data source

**Figure 1:**
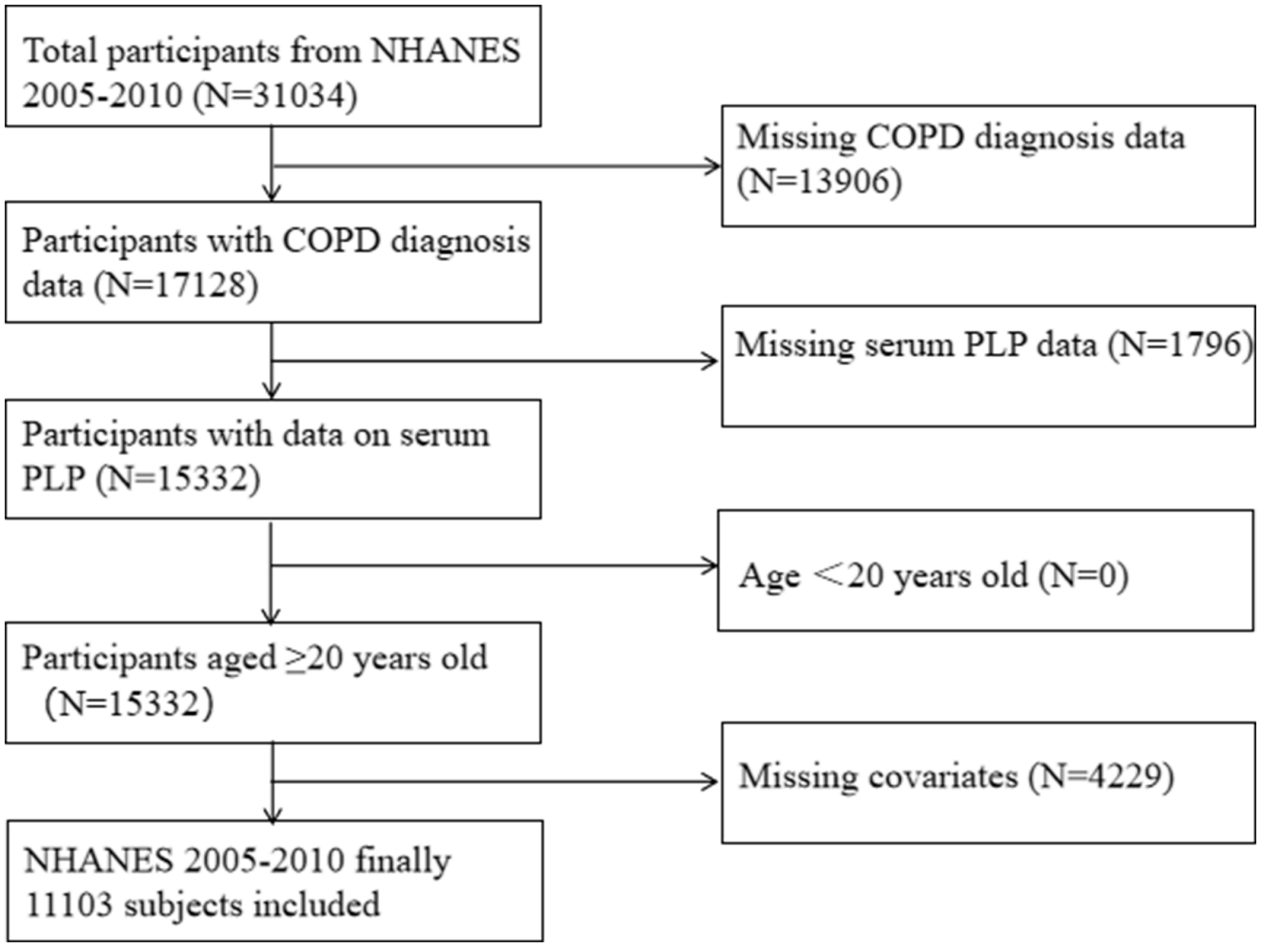
Flow chart of the study participants. NHANES, National Health and Nutrition Examination Survey. COPD, chronic obstructive pulmonary disease, PLP, 5′-pyridoxal phosphate.

The data for this study comes from three cycles of NHANES database, 2005–2006, 2007–2008, and 2009–2010, including demographic data, examination data, laboratory data, and questionnaire data. During the three cycles of NHANES 2005–2010, a total of 31,034 participants were enrolled. After excluding participants who lacked COPD diagnosis (*n* = 13,906), serum PLP data (*n* = 1796), were under 20 years old (*n* = 0), and lacked covariates data (*n* = 4,229), 11,103 participants were ultimately included in this study to explore the association between serum PLP levels and COPD prevalence (Figure 1).

### Chronic obstructive pulmonary disease definition

In this study, the diagnosis of COPD mainly depends on the patients self-report. According to the questionnaire data in the NHANES database, if the participant’s answer to “Ever told you had emphysema” or “Ever told you had chronic bronchitis” is “YES,” then he is diagnosed as having COPD.

### Vitamin B6 measurement

The data of vitamin B6 levels come from the laboratory data of the NHANES database. National Center for Environment Health (NCEH) or Center for Disease Control (CDC) used high - performance liquid chromatography (HPLC) method to measure the levels of serum PLP.

### Covariates

The covariates included in this study include gender (male, female), age (years), race (Mexican American, other Hispanic, Non-Hispanic White, Non-Hispanic Black, other races), education level (under high school, high school or equivalent, above high school), marital status (married or living with partner, widowed/divorced/separated, never married), family poverty income ratio (PIR) (<1, ≧1), body mass index (BMI) (kg/m^2^), cotinine levels, alcohol (drinks/never), history of hypertension, coronary heart disease (CHD), and diabetes. The levels of cotinine were divided into three groups: less than limit of detection (LOD), LOD-10, and above 10 ng/mL to evaluate the smoking status of participants. The alcohol consumption status is determined based on whether the participants drank alcohol at least once a month. The diagnosis of hypertension was based on the participant’s three times average systolic blood pressure greater than or equal to 140 mmHg, and/or diastolic blood pressure greater than or equal to 90 mmHg, or self-reported hypertension or taking antihypertension drugs. The diagnosis of diabetes was based on fasting blood glucose ≧7 mmol/L or glycosylated hemoglobin >6.5% or self-reported diabetes or taking hypoglycemic drugs or insulin.

### Statistical analysis

This study used R (version 4.4.2) and Empowerstats (version 4.2) for statistical analysis. Continuous variables are represented by mean ± standard errors (SE), while categorical variables are represented by proportion and 95% confidence interval (CI). We conducted three models: model 1 (unadjusted model), model 2 (adjusted for age, gender, and race), model 3 adjusted for age, gender, race, education level, marital status, family PIR, alcohol, cotinine, BMI, history of diabetes, hypertension, and CHD. We used GLM to analyze the correlation between serum PLP levels and COPD prevalence. To reduce the influence of confounding factors and evaluate the robustness of the associations, subgroup analysis and interaction tests were used to access the relationship between serum PLP levels and COPD prevalence across different groups. We used RCS curve and threshold effect analysis to explore the potential nonlinear relationship between serum PLP levels and COPD prevalence. Finally, we converted PLP into a binary variable based on the results of threshold effect analysis, and used weighted logistic regression analysis to evaluate the robustness of the relationship between serum PLP levels and COPD prevalence again.

## Results

### Baseline characteristics

In this study, we included a total of 11,103 participants with an average age of 48.79 years, including 5,882 males. We grouped according to the quartile range of serum PLP levels. As shown in Table 1, high-income males who are Non-Hispanic White, highly educated, lower BMI values, and have lower cotinine levels have higher levels of serum PLP, while participants with low serum PLP levels are more likely to exhibit advanced age, obesity, and low income levels. Meanwhile, participants with history of hypertension, coronary heart disease, and diabetes tended to have lower levels of serum PLP. It is worth noting that serum PLP levels in COPD formers are significantly lower than those in non-COPD formers.

**Table 1:** Base characteristics of participants in the NHNAES 2005–2010.

Variables	PLP (nmol/L)				*p*-value
	Q1	Q2	Q3	Q4	
	18.844 ± 0.128	34.876 ± 0.136	58.671 ± 0.199	173.894 ± 3.845	
Age (years)	48.157 ± 0.497	44.601 ± 0.411	45.178 ± 0.394	47.365 ± 0.629	<0.001
BMI(kg/m^2^)	30.684 ± 0.210	29.093 ± 0.175	28.303 ± 0.177	27.148 ± 0.159	<0.001
Gender (%)					<0.001
Male	38.937	51.345	59.88	56.332	
Female	61.063	48.655	40.12	46.668	
Race (%)					<0.001
Mexican American	6.536	9.085	8.794	5.844	
Other Hispanic	3.168	4.561	4.107	3.773	
Non-Hispanic White	72.015	69.672	74.103	79.431	
Non-Hispanic Black	14.517	11.13	7.911	6.476	
Other races	4.064	5.552	5.086	4.476	
Educational level (%)					<0.001
Under high school	22.677	19.773	15.478	11.27	
High school or equivalent	29.73	24.187	23.026	19.701	
Above high school	47.76	56.04	61.496	69.029	
Marital status (%)					<0.001
Married or Living with partner	60.331	65.481	66.893	69.009	
Widowed/Divorced/Separated	24.484	18.005	16.049	15.77	
Never married	15.185	16.514	17.058	15.221	
Family PIR (%)					<0.001
<1	17.718	13.307	10.83	7.594	
≥ 1	82.282	86.693	89.17	92.406	
Cotinine (%)					<0.001
< LOD	14.051	15.848	19.16	25.314	
LOD ~ 10	44.215	51.002	55.968	57.024	
>10	41.734	33.15	24.871	17.662	
Alcohol (%)					0.278
Drinks	3.552	3.369	3.311	4.394	
Never	96.448	96.631	96.689	95.606	
Hypertension (%)					<0.001
Yes	42.553	32.482	32.288	31.945	
No	57.447	67.518	67.712	68.055	
Diabetes (%)					<0.001
Yes	15.777	9.745	8.847	6.985	
No	84.223	90.255	91.153	93.015	
CHD (%)					0.008
Yes	4.449	3.302	2.423	2.982	
No	95.551	96.698	97.577	97.018	
COPD (%)					<0.001
Yes	11.376	6.9	4.999	5.21	
No	88.624	93.1	95.001	94.79	

Categorized based on serum PLP quartiles. Mean ± SE for continuous variables: the p value was calculated by the weighted linear regression model; (%) for categorical variables: the P value was calculated by the weighted chi-square test. PLP, 5′-pyridoxal phosphate, BMI, body mass index, PIR, poverty income ratio, LOD, limit of detection, CHD, coronary heart disease, COPD, chronic obstructive pulmonary disease.

### The association between serum PLP levels and COPD prevalence

The generalized linear model was used to discover the correlation between serum PLP levels and COPD prevalence. We constructed three models, namely the unadjusted model (model 1), the model adjusted for age, race, and gender (model 2), and the model adjusted for all relevant covariates (model 3). The results showed a significant association between serum PLP levels and COPD prevalence (OR = 0.999 95%CI: 0.996–0.999 *p* = 0.012). However, after adjusting for all related covariates, this relationship was not significant. We conducted further grouping analysis based on the quartiles of serum PLP levels. The results showed that compared with the Q2 group with lower serum PLP levels, higher serum PLP levels of Q3 and Q4 group were associated with lower COPD prevalence. Nevertheless, the relationship between serum PLP levels and COPD prevalence is not completely negatively correlated. When the serum PLP level is within the Q3 range, the prevalence of COPD reaches the lowest value. However, when the serum PLP level continues to rise to Q4 level, the prevalence of COPD is instead slightly higher than before (Table 2).

**Table 2:** Weighted logistic regression models for the association between serum PLP levels and COPD prevalence.

Characteristics	Model 1 OR (95% CI) *p*	Model 2 OR (95% CI) *p*	Model 3 OR (95% CI) *p*
PLP	0.998 (0.996, 1.000) 0.028	0.997 (0.996, 0.999) 0.012	0.999 (0.998, 1.001) 0.430
PLP (quartiles)			
Q1	ref	ref	ref
Q2	0.577 (0.447, 0.746) <0.001	0.653 (0.504, 0.846) 0.003	0.816 (0.623, 1.068) 0.150
Q3	0.410 (0.327, 0.513) <0.001	0.456 (0.360, 0.879) <0.001	0.646 (0.494, 0.845) 0.004
Q4	0.428 (0.340, 0.539) <0.001	0.429 (0.338, 0.545) <0.001	0.690 (0.538, 0.886) 0.007

Model 1: variables were not adjusted. Model 2: adjustments were made to age, gender, and race. Model 3: age, gender, race, educational level, marital status, family PIR, alcohol, cotinine, BMI, history of diabetes, hypertension, and CHD. PLP, 5′-pyridoxal phosphate, COPD, chronic obstructive pulmonary disease, OR, odds ratio, PIR, poverty income ratio, BMI, body mass index, CHD, coronary heart disease.

### Subgroup analysis and interaction effect tests

To reduce the influence of confounding factors and evaluate the robustness of the relationship between serum PLP levels and COPD prevalence among different groups, in order to identify potential population differences, we conducted multiple-subgroup analysis and interaction tests. The results showed that the relationship between serum PLP levels and COPD prevalence remained stable across all the subgroups, which confirmed the stability of the relationship between serum PLP levels and COPD prevalence (Table 3).

**Table 3:** Subgroup analysis and interaction effect tests for the association between serum PLP levels and COPD prevalence.

Subgroup analysis	COPD, OR(95% CI), *p*- value	*p* for interaction
Gender		0.959
Male	0.999 (0.997, 1.001) 0.538	
Female	0.999 (0.997, 1.002) 0.615	
Age (years)		0.612
< 60	1.000 (0.998, 1.002) 0.795	
≥ 60	0.999 (0.997, 1.001) 0.341	
Family PIR		0.784
<1	1.000 (0.996, 1.003) 0.924	
≥ 1	0.999 (0.998, 1.001) 0.379	
BMI (kg/m^2^)		0.241
< 28	1.000 (0.998, 1.002) 0.998	
≥ 28	0.998 (0.996, 1.001) 0.162	
Cotinine (ng/mL)		0.326
< LOD	1.001 (0.998, 1.003) 0.493	
LOD ~ 10	0.999 (0.998, 1.001) 0.551	
>10	0.998 (0.995, 1.001) 0.170	
Alcohol (%)		0.254
Yes	0.989 (0.974, 1.005) 0.200	
No	0.999 (0.997, 1.000) 0.130	
Hypertension (%)		0.237
Yes	0.999 (0.997, 1.000) 0.153	
No	1.000 (0.998, 1.002) 0.956	
Diabetes (%)		0.488
Yes	0.998 (0.992, 1.003) 0.393	
No	1.000 (0.998, 1.001) 0.590	
CHD (%)		0.724
Yes	0.998 (0.992, 1.005) 0.606	
No	1.000 (0.998, 1.001) 0.522	

COPD, chronic obstructive pulmonary disease, OR, odds ratio, PIR, poverty income ratio, BMI, body mass index.

### Restricted cubic spline curves and threshold effect analysis

**Figure 2:**
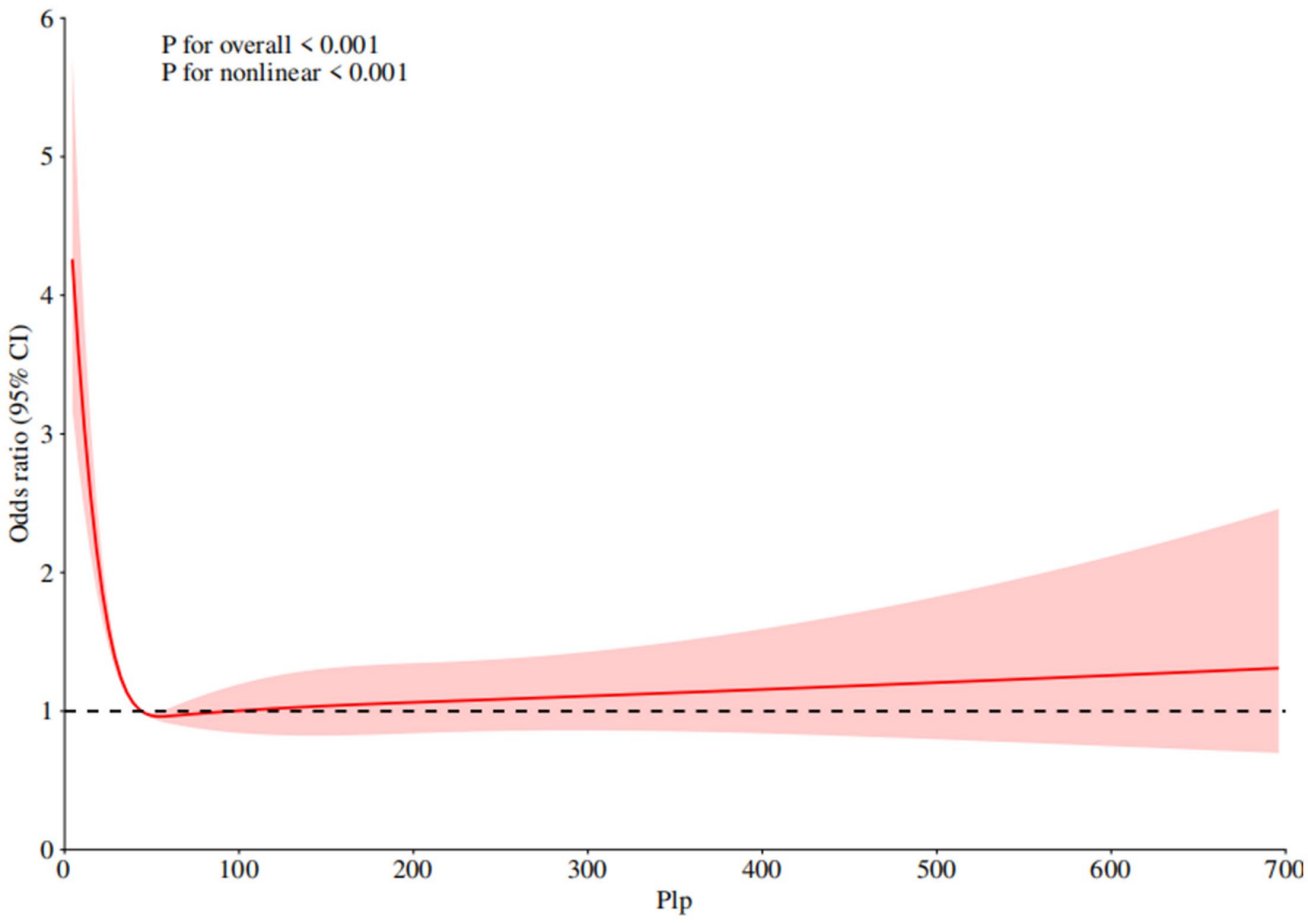
Non-linear relationship between serum PLP levels and COPD prevalence. RCS regression was adjusted for age, gender, race, education level, marital status, family PIR, alcohol, cotinine, BMI, history of diabetes, hypertension, and CHD. PLP, 5′-pyridoxal phosphate, COPD, chronic obstructive pulmonary disease, PIR, poverty income ratio, BMI, body mass index, CHD, coronary heart disease.

The RCS curve was used to explore the potential non-linear relationship between serum PLP levels and COPD prevalence. As shown in Figure 2, there is a “L”-shaped non-linear relationship between serum PLP levels and COPD prevalence (*p* for overall <0.001, *p* for nonlinear <0.001). When serum PLP is less than the threshold point, the OR of COPD prevalence significantly decrease with increasing serum PLP levels. When serum PLP levels reach a certain threshold, the prevalence of COPD will not significantly decrease with increasing serum PLP levels. Further analysis of the threshold effect presented a curve point at 43.3 (Table 4).

**Table 4:** The threshold effect analysis of serum PLP levels and COPD prevalence.

	OR (95% CI), *p*- value
Fitting by the standard linear model	0.999 (0.998, 1.000) 0.258
Fitting by the two-piecewise linear model	
Inflection point	43.3
Serum PLP < 43.3	0.983 (0.976, 0.990) <0.001
Serum PLP > 43.3	1.001 (1.000, 1.002) 0.243
p for log-likelihood ratio	<0.001

PLP, 5′-pyridoxal phosphate, COPD, chronic obstructive pulmonary disease, OR, odds ratio.

## Discussion

This cross-sectional study analyzed the relationship between serum PLP levels and COPD prevalence using various methods including generalized linear modeling, subgroup analysis, RCS curves, threshold effect analysis, and sensitivity analysis. The results showed a non-linear relationship between serum PLP levels and COPD prevalence.

Vitamin B6 is an essential trace element for the human body. Severe vitamin B6 deficiency can manifest as clinical symptoms and signs such as glossitis, rash, sensory abnormalities, anemia, and even epileptic seizures (18). However, due to the controversy over the critical point of serum PLP levels, some scholars believe that 20 nmol/L should be used as the critical point, while others believe that vitamin B6 should be confirmed to reach the edge state at a level less than 30 nmol/L (19, 20). Therefore, the definition of vitamin B6 deficiency is still not widely agreed upon. Nevertheless, many relevant studies have shown that, in addition to the severe lack of vitamin B6 increasing the risk of some chronic disease, even a mild deficiency of vitamin B6 deficiency has been confirmed to increase the risk of diabetes, cardiovascular disease (CVD), arthritis, chronic inflammatory bowel disease and cancer (21–24).

COPD, being a prevalent and preventable/treatable respiratory aliment, has posed a huge threat to people’s physical and mental well-being. At present, its treatment measures mainly include antibiotics, bronchodilators, and symptomatic therapy such as oxygen therapy. Regrettably, the current treatment status is not optimistic, with a persistently elevated mortality rate. Previously, some scholars have conducted research on the relationship between vitamin B6 and the complications of COPD. A case–control cross-sectional study showed that vitamin B6 deficiency can lead to hyperhomocysteinemia in COPD patients, thereby increasing the risk of arteriovenous thrombosis in COPD patients (25). Simultaneously, another investigation has found a negative correlation between the intake of vitamin B6 and the risk of frailty in COPD patients, and more intake of vitamin B6 may reduce the risk of concurrent frailty (13). The intake of vitamin B6 in COPD patients is significantly lower than that in healthy individuals (26).

It is worth noting that there is still a paucity of research regarding the association between vitamin B6 and the risk of COPD. This study found a dose-response relationship between serum PLP levels and COPD prevalence, confirming that serum PLP levels affect the prevalence of COPD. We speculate that this may be related to the important anti-inflammatory and antioxidant effects of vitamin B6, and a lack of vitamin B6 can promote inflammatory reactions and oxidative stress, thereby leading to the occurrence and development of COPD. The specific mechanism between serum PLP levels and COPD prevalence still requires further investigation.

This study is the first to analyze the non-linear relationship between serum PLP levels and COPD prevalence. Our study encompassed a total of 11,103 participants, of whom 830 were diagnosed with COPD. Consistent with previous studies, it was found that populations in low-income areas often have lower serum PLP levels (27, 28). At the same time, we discovered that people with low PLP levels tend to be older, more obese, and have higher incidence rate of hypertension, diabetes and coronary heart disease. Weighted logistic regression analysis showed that higher serum PLP levels were associated with lower prevalence of COPD. Additionally, we found that a serum PLP level of 43.3 nmol/L is the critical point in the study of COPD risk. When the serum PLP level is below 43.3 nmol/L, the risk of COPD is significantly increased. When the serum PLP level exceeds the cut-off point of 43.3, the prevalence of COPD does not remain completely unchanged in a straight line but shows a slowly increasing trend as the serum PLP rises. Although this trend is not as obvious as when the serum PLP level is less than 43.3, it cannot be ignored. This also suggests that an excessively high serum PLP level is related to an increased prevalence of COPD.

### Strengths and limitations

This study is the first to explore the relationship between serum PLP levels and COPD prevalence, and multiple adjustments were made to related covariates to ensure the robustness of the results of this study. However, it is necessary to acknowledge that this study still has certain limitations. Firstly, due to differences in individual conversion rates of vitamin precursors, the accuracy of measuring serum active vitamin B6 remains controversial. Secondly, this study is based on the population of the United States, and whether the results are applicable to other populations is still unknown. Thirdly, we cannot rule out the influence of other confounding factors that were not included in this study on the results. Finally, this study used the data from NHANES database from 2015 to 2020 as the research sample. These data may lack timeliness.

## Conclusion

This cross-sectional study confirms that higher serum PLP levels are associated with a reduced prevalence of COPD, and there is a non-linear relationship between serum PLP levels and COPD prevalence. 43.3 nmol/L is a crucial cut-off point. When the serum PLP level is below 43.3 nmol/L, the prevalence of COPD shows a significant upward trend. This non-linear relationship may provide new directions for thinking and research entry points for exploring the internal mechanism and potential intervention targets of the relationship between serum PLP levels and COPD prevalence, and also lay a theoretical foundation for future COPD prevention and treatment strategies based on the regulation of serum PLP levels.

## Data Availability

The original contributions presented in the study are included in the article/supplementary material, further inquiries can be directed to the corresponding authors.
